# The Resident Medical Officer of the Future

**Published:** 1912-05-18

**Authors:** 


### THE RESIDENT MEDICAL OFFICER OF THE FUTURE.

In our issue of April 27 we published an interview with an alienist holding an authoritative position, the subject of the interview being, of all things in the world, kitchen utensils; and we know our gentle readers, and, as Byron says, our "still gentler" subscribers, agree in thinking his remarks interesting and profitable. Dr. Jones' knowledge of pots and pans is in reality a sign of the new times in the whole medical world. Imperative in mental cases, institutional treatment of the sick generally on a large scale has been seen to be a good thing. Individual prejudice inspired by fear of loss of privacy, which was about the only thing against it, has been broken down by experience, and by the increasing social interdependence which marks the new century. It is found to be economical and vastly more efficient than domiciliary attendance. It disciplines the patient usefully, obviating the tendency to selfishness that assails the home invalid. It spares that invalid's relatives an extra burden over and above their ordinary work in the world. But large medical institutions to be really successful want all-round direction, as devoted as it is unfettered. Happily versatility is no uncommon quality in our practical Anglo-Saxon race, or in the sturdy middle-class calling of medicine. With competence as overseer and as (much-abused word!) organiser, professional knowledge and research of a high order are not at all incompatible. The class of man who combines these attributes is the one likely to be increasingly required in the near future. He need not have an independent income, or be fashionably connected, or do deep obeisance to professional hierarchs, but he must, as the pithy Americanism has it, be able to make good all round. And if he fails in this he must go; we have seen enough of the evils of over-security of tenure of office. These posts will surely attract some of our best men. Opportunity of abundant clinical material, scope for initiative and individuality, freedom from weary waiting, from "placebos" and "conversation calls," from social backstairs, from snubbing by unsuccessful, embittered seniors, from pinching to pay the rent of chambers in which one sits alone all day—these are all undeniable boons. In the past, perhaps—with, of course, honourable exceptions—institutional medical officers have been recruited too much from the type that considers life work finished with the gaining of the higher degree that brings the appointment, except for a prudent performance of routine. An ideal that might well be held up to medical aspirants when October comes round again, is to be a real social servant of high intellectual grade, rewarded, not with a fortune and notice in mediocre lay newspapers, but with a competence and the respect of true critics.

